# Enhanced sensitivity to cisplatin and gemcitabine in *Brca1*-deficient murine mammary epithelial cells

**DOI:** 10.1186/1471-2210-11-7

**Published:** 2011-07-19

**Authors:** Elizabeth Alli, Vandana B Sharma, Anne-Renee Hartman, Patrick S Lin, Lisa McPherson, James M Ford

**Affiliations:** 1 Department of Medicine, Division of Oncology, Stanford University School of Medicine, Center for Clinical Sciences Research, Stanford, CA 94305, USA; 2 Department of Genetics, Stanford University School of Medicine, Stanford, CA 94305, USA

## Abstract

**Background:**

Breast cancers due to germline mutations or altered expression of the *BRCA1 *gene associate with an aggressive clinical course and frequently exhibit a "triple-negative" phenotype, i.e. lack of expression of the estrogen and progesterone hormone receptors and lack of overexpression of the HER2/NEU oncogene, thereby rendering them relatively insensitive to hormonal manipulation and targeted HER2 therapy, respectively. BRCA1 plays a role in multiple DNA repair pathways, and thus, when mutated, results in sensitivity to certain DNA damaging drugs.

**Results:**

Here, we used a *Brca1 *murine mammary epithelial cell (MMEC) model to examine the effect of loss of *Brca1 *on cellular sensitivity to various chemotherapy drugs. To explore novel therapeutic strategies, we included DNA damaging and non-DNA damaging drugs whose mechanisms are dependent and independent of DNA repair, respectively, and drugs that are used in standard and non-standard lines of therapy for breast cancer. To understand the cellular mechanism, we also determined the role that DNA repair plays in sensitivity to these drugs. We found that cisplatin and gemcitabine had the greatest specific therapeutic benefit to *Brca1*-deficient MMECs, and that when used in combination produced a synergistic effect. This sensitivity may be attributed in part to defective NER, which is one of the DNA repair pathways normally responsible for repairing DNA adducts produced by cisplatin and is shown in this study to be defective in *Brca1*-deficient MMECs. *Brca1*-deficient MMECs were not differentially sensitive to the standard breast cancer chemotherapy drugs doxorubicin, docetaxel or 5-FU.

**Conclusions:**

Both cisplatin and gemcitabine should be explored in clinical trials for first line regimens for BRCA1-associated and triple-negative breast cancer.

## Background

Inheritance of a mutation in the *BRCA1 *gene confers a 45-65% average lifetime risk for developing breast cancer and an increased risk for developing ovarian cancer [1]. While germline mutations in *BRCA1 *account for 5% of breast cancer cases, evidence suggests that epigenetic silencing of *BRCA1 *by promoter hypermethylation and other mechanisms may contribute to up to 30% of sporadic breast cancers [2-7]. *BRCA1*-associated breast cancers have a characteristic phenotype; in general, these tumors have a high mitotic index, contain p53 mutations, and often exhibit a triple-negative phenotype (i.e. lack of expression of estrogen and progesterone receptors and lack of overexpression of the HER2/NEU oncogene) [8,9]. This triple-negative status renders *BRCA1*-associated cancers insensitive to hormonal manipulation or targeted therapy with trastuzumab, respectively. With the exception of PARP inhibitors, an investigational therapeutic strategy for BRCA-deficient cancers [10], empirically chosen cytotoxic chemotherapy is the primary option for treating patients with BRCA1-associated and triple-negative breast cancer.

BRCA1 plays multiple roles in DNA damage response pathways. BRCA1 has a well-established role in DNA double-strand break repair [11]. More recently our lab has shown that BRCA1 is involved in DNA base-excision repair (BER) [12] and nucleotide-excision repair (NER) [13,14]. BER repairs single base-pair lesions that are typically induced by endogenous agents, such as oxidative byproducts of normal cellular metabolism. NER functions to repair bulky lesions or DNA adducts induced by exogenous means such as ultraviolet (UV)-irradiation, carcinogens including polyaromatic hydrocarbons and tobacco, and certain chemotherapy agents such as cisplatin. NER can be subdivided into two genetically distinct subpathways: global genomic repair (GGR) that removes lesions from the whole genome and transcription-coupled repair (TCR) that removes lesions from actively transcribed DNA. We have shown in human tumor cells that BRCA1 directly affects the GGR subpathway of NER, and that this function may occur through transcriptional regulation of NER genes involved in the recognition of adducts in genomic DNA, including XPC and DDB2 (the genes mutated in xeroderma pigmentosum complementation groups C and E, respectively) [13].

Cellular characteristics that contribute to carcinogenesis, such as defects found in DNA repair pathways, may be exploited for cancer therapy. For example, cancer cells deficient in BRCA1 tend to exhibit defective DNA repair, and in turn, are sensitive to drugs such as mitomycin C and cisplatin, which induce intrastrand and interstrand DNA crosslinks, stalled replication forks, and DNA double-strand breaks [15-20], and PARP inhibitors, which through a synthetic lethal mechanism further inhibit DNA repair mechanisms and promote cytotoxicity [21,22].

Here, we used an isogenic *Brca1 *murine mammary epithelial cell (MMEC) model to examine the specific effect of loss of *Brca1 *on cellular sensitivity to various chemotherapeutic agents in a manner beyond that achievable in less well-characterized human tumor cell lines. We included DNA damaging and non-DNA damaging drugs whose mechanisms are dependent and independent of DNA repair, respectively, and drugs that are used in standard and non-standard lines of therapy for breast cancer.

## Methods

### Cell Lines

*Brca1*^+/+ ^and *Brca1*^-/- ^MMECs were kindly provided by the laboratory of Kenneth H. Cowan (Eppley Institute for Research in Cancer and Allied Diseases, University of Nebraska Medical Center) and were cultured as previously described [23]. MMECs were isolated from *Brca1*^fl/fl ^mice [24]. These mice carry loxP sites flanking exon 11 of the *Brca1 *gene and develop normally. *Brca1*^fl/fl ^MMECs were infected with an HPV-16E6 (Neo^+^) retrovirus to inhibit p53 function and immortalize the cells. *Brca1*^-/- ^MMECs were generated by deleting exon 11 of *Brca1 *following transfection with pBabe-Cre (Puro^+^) retrovirus.

### Real-time RT-PCR (RT-qPCR)

Total RNA was isolated and purified using RNeasy Protect Mini Kit (Qiagen) with the following modifications. Cells were homogenized using the QIAshredder column (Qiagen) and the resulting lysates treated with RNase-Free DNase (Qiagen) to remove genomic DNA. Total RNA (2.5 μg) from each sample was reverse transcribed using SuperScript™ III First-Strand Synthesis System (Invitrogen) to create cDNA libraries. The Platinum^® ^SYBR^® ^Green qPCR SuperMix-UDG (Invitrogen) was used for PCR of cDNA samples in a protocol consisting of 50 cycles of denaturation (95°C for 15 sec), primer annealing (57°C for 30 sec), and primer extension (72°C for 30 sec) using an ABI PRISM 7900 Sequence Detection System (Applied Biosystems). For calibration and generation of standard curves, we used cDNA from mouse embryo fibroblasts as reference standards [25]. All reactions were carried out in triplicate with minimal Ct variability seen. The transcript level of each gene was normalized to that of *Gapdh *and expressed as fold induction over 0-hour reference level to examine UV damage-inducible transcripts and over untreated control to examine drug-inducible transcripts. The mouse *Ddb2 *primers used were 5'-GCCGATACCCAGATCCTAATCTT-3' and 5'-ACACATCATCTTCCCTGAGCTTC-3'. The mouse *Xpc *primers used were 5'-ATCATTCCAATTCGCTTTACCAA-3' and 5'-GTTCCGATGAACCACTTTACCAG-3'. The mouse *Xpa *primers used were 5'-CACCAAAGGTGGCTTCATTTTAG-3' and 5'-TGGTGTAATCAAACTCCATGACG-3'. The mouse *Gapdh *primers used were 5'-GGAGAAACCTGCCAAGTATGATG-3' and 5'GACAACCTGGTCCTCAGTGTAGC-3'.

### GGR Assay

Repair of DNA adducts, cyclobutane pyrimidine dimers (CPDs) and 6-4 photoproducts (6-4PPs), from total genomic DNA at different times following UV-irradiation was measured using an immunoslot blot assay as previously described [13,26]. To control for replication, ^3^H-thymidine labeled cells were used. Monoclonal antibodies specific for either CPDs (1:1000) or 6-4PPs (1:500) were kindly supplied by Toshio Mori (Nara Medical University, Japan). Genomic DNA from unirradiated cells was loaded as a control for nonspecific antibody binding. Data from triplicate DNA samples from three different biological experiments were averaged and normalized to the unrepaired damaged control (i.e. UV = 10 J/m^2^, Time = 0). Statistical analysis of differences in DNA repair curves due to expression of *Brca1 *were performed using the unpaired T-test.

### TCR Assay

To determine the rate of removal of adducts from the transcribed strand of a specific gene fragment, strand-specific RNA probes were used to evaluate the frequency of CPDs in a 14-kb *Bam*H1 restriction fragment spanning the central region of the mouse *Dhfr *gene, as previously described [27,28]. Cells were irradiated with 10 J/m^2 ^of UV-C, lysed immediately for an initial sample (time = 0), or incubated for up to 24 hrs to allow lesion repair. The frequency of induction and rate of removal of CPDs from the transcribed strand and non-transcribed strand of the *Dhfr *gene was measured by treating purified *Bam*HI-digested DNA with bacteriophage T4 endonuclease V (generously supplied by R. Stephen Lloyd, Oregon Health Sciences University), and then quantifying the reappearance of the full-length restriction fragments in DNA from cells allowed various times to remove the lesions using denaturing electrophoresis and Southern blotting.

### Cell Sensitivity Assays

For UV sensitivity, cells were plated in 96 well plates at a density of 10^3 ^cells/well in triplicate and allowed to attach overnight. Cells were then washed with PBS, exposed to UV-C irradiation at doses of 0, 5, 10, 20, and 30 J/m^2^, and allowed to recover for 48 hours. For drug sensitivity, cells were plated in triplicate and allowed to attach overnight. Cells were treated with increasing concentrations of doxorubicin, 5-FU, or paclitaxel for 48 hours, or cisplatin (Sigma-Aldrich), carboplatin, oxaliplatin, or gemcitabine for 72 hours. Drugs were provided by the Stanford Cancer Center unless otherwise indicated. Incubation times were determined to be the shortest number of 24 hour periods that produced a full dose-response curve and were carried out at 37°C and 5% CO_2_. Media was then removed and replaced with fresh media containing 1 mg/ml 3-(4,5-dimethylthiazol-2-yl)-2,5-diphenyltetrazolium bromide (MTT) solution until formation of formazan crystals (~3 hours). The MTT formazan crystals were dissolved in DMSO (200 μl/well) and glycine buffer (25 μl/well). Absorbance was measured at 570 nm with a VERSAmax microplate reader (Molecular Devices) and a logarithmic plot of absorbance versus UV dose or drug concentration recorded. Cell viability was expressed as the ratio of the treated cells to that of the untreated controls at each dose or concentration. The IC_50 _value for each cell line was determined using SoftMax^® ^Pro software (Molecular Devices) and statistical significance calculated by students t-test using the average IC_50 _values from multiple independent experiments.

### Combination Treatment

Cells were treated with cisplatin and gemcitabine alone and in various dose combinations for 48 hours and then subjected to the MTT assay described above. Isobologram analysis differentiated between antagonism, synergism, and additive effects as previously described [29]. Combination index was determined as described by Chou and Talalay [30].

### Alkaline Comet Assay

At 24 hours following treatment with drug, cells were subjected to the alkaline comet assay for the detection of DNA strand breaks as previously described [31]. Briefly, cells were embedded at low density onto comet slides, lysed, exposed to alkaline conditions to denature DNA, and subjected to electrophoresis. DNA was stained with SYBR^® ^green and visualized by fluorescent microscopy as a comet in shape. The percentage of DNA in the comet tails, i.e. DNA damage, was calculated using CometScore software (TriTek Corporation).

## Results

### Characterization of the Cellular System

BRCA1-associated cancers, including hereditary breast cancers due to germline mutations and sporadic breast cancers associated with promoter hypermethylation, have reduced or complete loss of expression of BRCA1 protein, and are frequently accompanied by mutations in *TP53 *[2,8,32,33]. Therefore, to study the effect of loss of BRCA1 expression on chemosensitivity to DNA repair- dependent and independent drugs, we used *Brca1*^+/+ ^and *Brca1*^-/- ^MMECs that were generated by disrupting the *Brca1 *gene in MMECs immortalized and p53-inactivated by infection with HPV-16E6. These cells have previously been shown to have undergone homozygous deletion of *Brca1 *exon 11 and to have lost expression by RT-PCR, Northern and Western blotting [23]. RT-PCR confirmed the expression and loss of expression of *Brca1 *in *Brca1*^+/+ ^and *Brca1*^-/- ^MMECs, respectively (data not shown). The *Brca1^-/- ^*MMECs have also been reported to harbor defective DNA base-excision repair [12] as well as increased genetic instability [34] compared to *Brca1*^+/+ ^MMECs, which is typical of *BRCA1*-mutant breast cancer cells. Furthermore, the *Brca1*^+/+ ^and *Brca1*^-/- ^MMECs showed similar proliferation rates (data not shown), which allowed for direct comparison of sensitivity to various drugs.

### Effect of Loss of *Brca1 *on Sensitivity to DNA Damaging Agents

**Figure 1:**
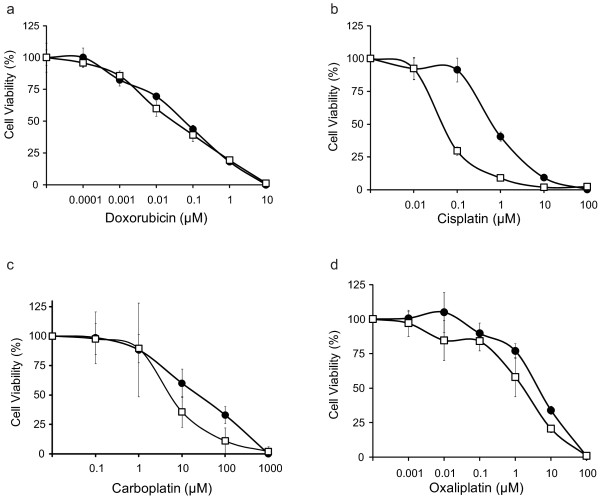
**Effect of BRCA1-deficiency on Sensitivity to DNA Damaging Agents**. *Brca1*^+/+ ^(black circle) and *Brca1*^-/- ^(white square) cells were analyzed for cellular sensitivity to (a) doxorubicin (b) cisplatin (c) carboplatin or (d) oxaliplatin by MTT assay. Each data point represents the average of triplicate readings ± S.D. Graphs are representative of at least three independent experiments.

We examined the effect of loss of *Brca1 *on sensitivity to DNA damaging agents, including doxorubicin, cisplatin, carboplatin, and oxaliplatin. Doxorubicin is an anthracycline that inhibits topoisomerase II and thereby produces DNA double-strand breaks; it is commonly used in the treatment of breast cancer. Cisplatin, carboplatin, and oxaliplatin are platinum agents, which induce intra- and inter-strand DNA crosslinks that are typically repaired by NER, or when left unrepaired, convert to DNA double-strand breaks. Following treatment with increasing concentrations of each drug, *Brca1*^+/+ ^and *Brca1*^-/- ^MMECs were analyzed for sensitivity by MTT assay. *Brca1*^+/+ ^and *Brca1*^-/- ^MMECs were similarly sensitive to doxorubicin (Figure 1a and Table 1; p = 0.3). However, *Brca1*^-/- ^MMECs were more sensitive than *Brca1*^+/+ ^MMECs to all of the platinum agents (Figure 1b-d and Table 1), with the greatest difference being observed for cisplatin. Specifically, loss of *Brca1 *associated with a 13-fold increase in sensitivity to cisplatin (Figure 1b; p = 0.001).

**Table 1:** IC_50 _Values for Chemotherapy Drugs

	*Brca1*^+/+ ^MMECs	*Brca1*^-/- ^MMECs	p-value
**DNA-damaging Drugs**	**(μM)**	**(μM)**	

Doxorubicin	0.06 ± 0.02	0.04 ± 0.02	0.3

Cisplatin	0.8 ± 0.1	0.06 ± 0.02	0.001**

Carboplatin	10 ± 4	6 ± 2	0.03*

Oxaliplatin	5 ± 1	2 ± 1	0.04*

**Taxanes**			

Paclitaxel	0.4 ± 0.3	0.08 ± 0.06	0.1

Docetaxel	0.001 ± 0.0009	0.002 ± 0.002	0.5

**Antimetabolites**			

5-FU	0.8 ± 0.5	4 ± 0.9	0.004**

Gemcitabine	0.05 ± 0.02	0.002 ± 0.	0.02*

**Highly statistically significant (p < 0.01)

*Statistically significant (p < 0.05)

### Effect of Loss of *Brca1 *on Sensitivity to Non-DNA Damaging Agents

**Figure 2:**
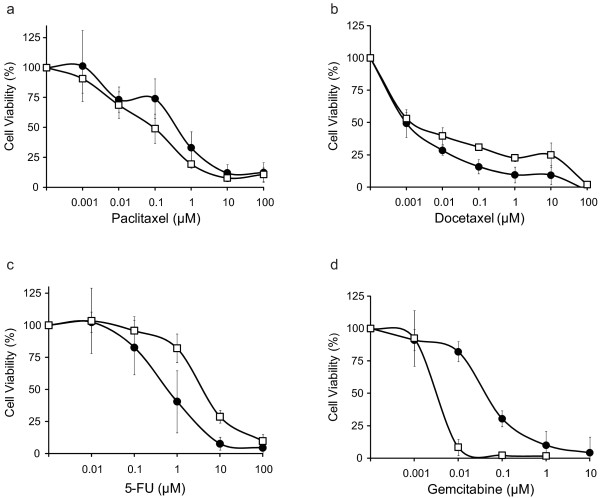
**Effect of *Brca1*-deficiency on Sensitivity to Non-DNA Damaging Agents**. *Brca1*^+/+ ^(black circle) and *Brca1*^-/- ^(white square) cells were analyzed for cellular sensitivity to taxanes, paclitaxel (a) or docetaxel (b), and to antimetabolites, 5-FU (c) or gemcitabine (d) by MTT assay. Each data point represents the average of triplicate readings ± S.D. Graphs are representative of at least three independent experiments.

We next examined the effect of loss of *Brca1 *on sensitivity to non-DNA damaging drugs thought to be independent of DNA repair function. *Brca1*^+/+ ^and *Brca1*^-/- ^MMECs were treated with increasing concentrations of taxanes (paclitaxel or docetaxel) or antimetabolites (5-FU or gemcitabine), and evaluated for cellular sensitivity by MTT assay. We found that *Brca1*^+/+ ^and *Brca1*^-/- ^MMECs were similarly sensitive to both paclitaxel (Figure 2a; p = 0.1) and docetaxel (Figure 2b; p = 0.5). Sensitivity to the antimetabolites, on the other hand, produced contrasting results. Compared to *Brca1*^+/+ ^MMECs, *Brca1*^-/- ^MMECs were 5-fold less sensitive to 5-FU (Figure 2c; p = 0.004), but 27-fold more sensitive to gemcitabine (Figure 2d; p = 0.02). Table 1 summarizes these data. Interestingly, loss of *Brca1 *associated with cellular sensitivity to gemcitabine, which unlike the taxanes and 5-FU, is not currently used among standard first lines of therapy for breast cancer.

### Combination Therapy in *Brca1*^+/+ ^and *Brca1*^-/- ^MMECs

**Figure 3:**
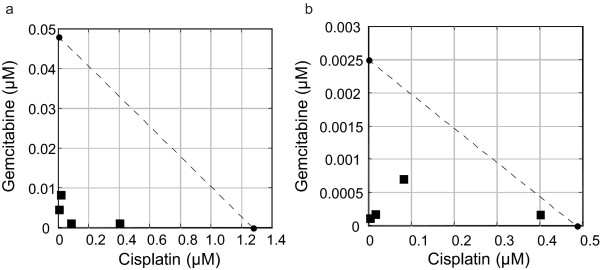
**Sensitivity to the Combination of Cisplatin and Gemcitabine**. *Brca1*^+/+ ^(a) and *Brca1*^-/- ^(b) MMECs were treated with cisplatin and gemcitabine either alone or in combination and analyzed for sensitivity by MTT assay. The IC_50 _values determined from treatment with cisplatin and gemcitabine alone were plotted as axial points (black circles) on a Cartesian plot to generate a line of additivity. The IC_50 _values for each combination of cisplatin and gemcitabine were then plotted as data points (black squares). Data points above the line of additivity represent an antagonistic effect, data points on the line of additivity represent an additive effect, and data points below the line additivity represent a synergistic effect. Data are representative of at least three independent experiments.

Our data has indicated that loss of *Brca1 *produced the greatest sensitivity to cisplatin (Figure 1b) and to gemcitabine (Figure 2d). Therefore, we next assessed the effect of these drugs used in combination by isobologram analysis and found that there was a synergistic effect between cisplatin and gemcitabine in both *Brca1*^+/+ ^and *Brca1*^-/- ^MMECs, but that the concentrations required to produce the synergistic effect in *Brca1*^-/- ^MMECs were much lower than those needed for *Brca1*^+/+ ^MMECs (Figure 3). The combination index (CI) for *Brca1*^+/+ ^and *Brca1*^-/- ^MMECs were 0.01 and 0.05, respectively, where CI < 1 is synergism, CI = 1 is additive, and CI > 1 is antagonism. Therefore, these data confirmed synergism between cisplatin and gemcitabine.

### Effect of Loss of *Brca1 *on NER

**Figure 4:**
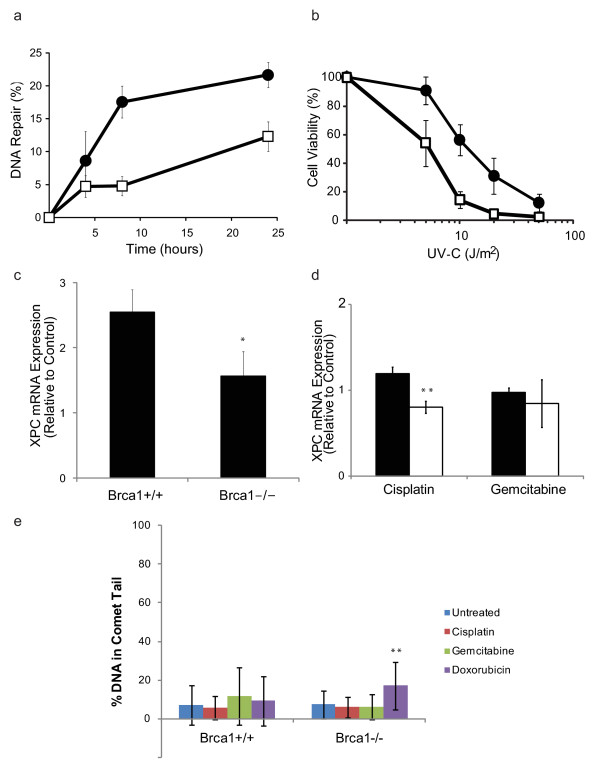
**Effect of *Brca1*-deficiency on NER and Double-strand DNA Break Repair**. In (a), GGR of CPDs in *Brca1*^+/+ ^(black circle) and *Brca1*^-/- ^(white square) cells was measured using an immunoslot blot assay. Cells were exposed to 10 J/m^2 ^UV-irradiation and collected at the indicated times. DNA repair was expressed as a percentage relative to control. Data from triplicate DNA samples from three different biological experiments were expressed as an average ± S.E.M. In (b), sensitivity to UV-irradiation was determined by MTT assay for *Brca1*^+/+ ^(black circle) and *Brca1*^-/- ^(white square) cells. In (c), damage-induced expression of *Xpc *mRNA, an NER gene involved in DNA damage recognition, in *Brca1*^+/+ ^and *Brca1*^-/- ^cells was measured using RT-qPCR. *Brca1*^+/+ ^and *Brca1*^-/- ^cells were exposed to 10 J/m^2 ^of UV and either harvested immediately (control) or incubated in media and harvested 24 h later. In (d), expression of *Xpc *mRNA following 24 hours of treatment with 0.1 μM cisplatin or 0.01 μM gemcitabine in *Brca1*^+/+ ^and *Brca1*^-/- ^cells was measured using RT-qPCR. Data were calculated relative to the untreated control and expressed as the average of three experiments ± S.E.M. In (e), DNA strand breaks were measured at 24 hours following treatment with 0.1 μM cisplatin, 0.01 μM gemcitabine, or 0.1 μM doxorubicin in *Brca1*^+/+ ^and *Brca1*^-/- ^cells using the alkaline comet assay. Comet tails indicate DNA damage. Unless indicated otherwise, data were expressed as an average of triplicate readings ± S.D. **, p < 0.01; *, p < 0.05.

Given that cisplatin produces lesions that are repaired by NER, we next examined the functionality of the NER pathway in *Brca1*^+/+ ^and *Brca1*^-/-^MMECs. Using an assay for GGR that measures the removal of UV-induced DNA lesions (CPDs and 6-4PPs), we found that repair of CPDs 24 hrs after UV irradiation decreased from 22 ± 2% in *Brca1*^+/+ ^MMECs to 12 ± 2% in *Brca1*^-/- ^MMECs (Figure 4a; p = 0.025). No difference was observed for 6-4PPs; both cell lines repaired nearly 100% of these adducts by 24 hrs (data not shown). Analysis of TCR showed no difference between *Brca1*^+/+ ^and *Brca1*^-/- ^MMECs with greater than 70% repair of CPDs in the transcribed strand of the mouse *dhfr *gene being observed at 24 hours (data not shown).

To investigate the biological consequence of the different abilities in GGR between *Brca1*^+/+ ^and *Brca1*^-/- ^MMECs, we examined cell survival after UV irradiation. As shown in Figure 4b, cell viability decreased in a dose-response manner in both *Brca1*^+/+ ^and *Brca1*^-/- ^MMECs following exposure to increasing amounts of UV irradiation. However, *Brca1*^-/- ^MMECs displayed a 3-fold increase in sensitivity to UV irradiation relative to that of *Brca1*^+/+ ^MMECs (p = 0.029).

To further explore the GGR defect in *Brca1*^-/- ^MMECs, we investigated whether loss of *Brca1 *may affect the expression of NER genes. We and others have shown that BRCA1 regulates the expression of human *DDB2 *and *XPC *[13,35], and the products of these genes are required for efficient GGR of CPDs in human cells [36]. Therefore, we evaluated the expression of *Ddb2 *and *Xpc *in *Brca1*^+/+ ^and *Brca1*^-/- ^MMECs by RT-qPCR under the same conditions as those used for the GGR assay. When compared to *Brca1*^+/+ ^MMECs, *Brca1*^-/- ^MMECs showed a statistically significant decrease in UV-induced expression of *Xpc *mRNA (p = 0.04) but not of *Ddb2 *mRNA (p = 0.8) (Figure 4c and data not shown).

We next examined the effect of loss of *Brca1 *on drug-induced expression of *Xpc*. Following treatment with untreated control, 0.1 μM cisplatin, or 0.01 μM gemcitabine for 24 hours, we analyzed levels of *Xpc *mRNA by RT-qPCR in *Brca1*^+/+ ^and *Brca1*^-/- ^MMECs. *Brca1*^-/- ^MMECs showed significantly less induction of *Xpc *mRNA following cisplatin treatment compared to *Brca1*^+/+ ^MMECs (p = 0.009; Figure 4d), whereas both *Brca1*^+/+ ^and *Brca1*^-/- ^MMECs showed no significant increase in *Xpc *mRNA expression following treatment with gemcitabine (p = 0.6; Figure 4d). Taken together, these data suggest that the increase in sensitivity to platinum agents observed due to the loss of *Brca1 *expression may be attributed in part to an attenuation of transcriptional regulation of *Xpc*, a DNA damage recognition gene, and a subsequent decrease in GGR function of the NER pathway.

### Effect of Loss of *Brca1 *on DNA Double-strand break repair

Finally, due to the well-established role for BRCA1 in DNA double-strand break repair, we examined the effect of loss of Brca1 on levels of DNA strand breaks following treatment with certain drugs. *Brca1*^+/+ ^and *Brca1*^-/- ^MMECs were left untreated (control) or treated with 0.1 μM cisplatin, 0.01 μM gemcitabine, or 0.1 μM doxorubicin, and then after 24 hours for repair, subjected to the alkaline comet assay (Figure 4e). We observed similar levels of DNA strand breaks following treatment with cisplatin or gemcitabine compared to control in both *Brca1*^+/+ ^(p = 0.5 and p = 0.08, respectively) and *Brca1*^-/- ^MMECs (p = 0.4 and p = 0.6, respectively). However, we found significantly greater levels of DNA strand breaks following treatment with doxorubicin compared to the untreated control in *Brca1*^-/- ^MMECs (p = 0.0004), but not in *Brca1*^+/+ ^MMECs (p = 0.4).

## Discussion

Loss of BRCA1 function plays a role in the development of a substantial number of breast cancers, including more than 50% of hereditary cases due to germline mutations [37] and up to 30% of sporadic cases through mechanisms of epigenetic silencing [4]. *BRCA1*-associated cancers are typically triple-negative in phenotype, correlate with a poor clinical outcome [38-41], and are in need of improved treatment options. BRCA1 functions in DNA damage response pathways, cell-cycle control, chromatin remodeling, transcription regulation, and various other cellular processes [42-45]. In this study, we evaluated the cellular sensitivity to drugs that are dependent and independent of DNA damage response pathways and analyzed the corresponding DNA repair status in *Brca1*^+/+ ^and *Brca1*^-/- ^MMECs. We used an isogenic cellular system to allow the direct comparison of sensitivity to various drugs and found that among the drugs used in this study, cisplatin and gemcitabine produced the greatest therapeutic benefit to *Brca1*-deficient MMECs, and that when used in combination, produced a synergistic effect. This sensitivity may be attributed in part to defective NER, which is one of the DNA repair pathways normally responsible for repairing DNA adducts generated by cisplatin and is shown in this study to be defective in *Brca1*-deficient MMECs.

Loss of *Brca1 *associates with sensitivity to platinum-based DNA-damaging agents. We found that the greatest therapeutic advantage to *Brca1*-deficient MMECs among the platinum agents to be with cisplatin, moreso than carboplatin or oxaliplatin. *Brca1*^-/- ^MMECs were 13-fold more sensitive to cisplatin than *Brca1*^+/+ ^MMECs (Figure 1b). While all platinum agents have the common ability to cross-link DNA, major differences occur in their mechanisms of action and resistance and in their stability (reviewed in [46-48]) that may explain the greater sensitivity to cisplatin compared to other members of the group. Consistent with our data, Sgagias et al. reported cisplatin sensitivity in these *Brca1*-deficient cells [23], and others have reported similar cisplatin sensitivity in other BRCA1-deficient cellular systems [15-20,49]. Platinum agents induce DNA adducts and double-strand breaks that are typically repaired by NER and homologous recombination (HR), respectively [50]. Loss of BRCA1 is generally believed to associate with cisplatin sensitivity due to compromised HR, resulting in unrepairable DNA double strand breaks and subsequent cell death. Under our experimental conditions, we observed no difference in the levels of DNA strand breaks following cisplatin treatment in Brcal-wild-type versus null cells (Figure 4e). However, cells derived from the same *Brca1 *mouse model as the MMECs that are described in this study have been shown to display genetic instability and sensitivity to agents that produce double-strand breaks [34], suggesting that these cells, like other BRCA1-deficient cells, display defective double-strand break repair. Therefore, the conditions for cisplatin treatment used in our experiments may have produced platinum -DNA adducts moreso than DNA double-strand breaks. We found that loss of *Brca1 *expression resulted in a defect in the GGR subpathway of NER. Specifically, we demonstrated that *Brca1*-deficient MMECs showed a reduced rate of GGR of CPDs (Figure 4a), significantly increased sensitivity to UV-irradiation (Figure 4b), and loss of *Xpc *transcriptional induction after DNA damage (Figure 4c). Similarly, *Brca1*-deficient MMECs exhibited sensitivity to platinum agents (Figure 1b-d) and a loss of *Xpc *transcriptional induction after cisplatin treatment (Figure 4d). These data are consistent with human studies showing that overexpression of human BRCA1 enhances GGR through transcriptional regulation of NER genes, *XPC, DDB2*, and *GADD45 *[13]. Therefore, loss of BRCA1 may also result in cisplatin sensitivity due to compromised NER. In support of this idea, both HR and NER have been described as mechanisms of resistance to cisplatin [50,51]. Furthermore, these same *Brca1*-deficient MMECs were more sensitive to inhibitors of PARP, a BER enzyme, and MMS, which produces lesions repaired by BER, and showed an aberrant response to oxidative stress that is consistent with a defect in BER [12,23]. Taken together, we propose that multiple repair pathways are likely to be responsible for BRCA1-mediated sensitivity to platinum agents, including NER and HR.

In contrast to cisplatin, loss of *Brca1 *did not affect sensitivity to the DNA-damaging agent doxorubicin. We found no difference in sensitivity between *Brca1*^+/+ ^and *Brca1*^-/- ^MMECs (Figure 1a). Doxorubicin intercalates within DNA and inhibits topoisomerase II, resulting in DNA double-strand breaks. Given that the response to doxorubicin is dependent in part on HR for the repair of double-strand breaks and the response to platinum agents is dependent on both HR and NER, our finding that *Brca1*-deficient cells were defective in the GGR subpathway of NER may explain the difference in sensitivity between these two types of DNA damaging agents. Furthermore, the lack of effect on sensitivity to doxorubicin in *Brca1*-deficient MMECs may be attributed to the fact that topoisomerase II unwinds DNA for transcription, and we found that *Brca1*-deficient cells were proficient in TCR (data not shown). We observed greater levels of DNA strand breaks in *Brca1*^-/- ^MMECs compared to *Brca1*^+/+ ^MMECs at 24 hours following doxorubicin treatment (Figure 4e). Therefore, the intercalating activity, which inhibits DNA replication and synthesis, may dominate over the topoisomerase II inhibiting activity of doxorubicin, and thus, eliminate the specificity for cell killing of *Brca1*-deficient cells. In support of this idea, Treszezamsky et al. found that due to compromised HR, BRCA1-deficent cells were sensitive to etoposide [52], which is a topoisomerase II poison but not a DNA intercalator. Therefore, both activities of doxorubicin may contribute to the comparable sensitivity for doxorubicin in *Brca1*^+/+ ^and *Brca1*^-/- ^MMECs.

Loss of *Brca1 *exhibits a variable response to antimetabolites. *Brca1*^-/- ^MMECs were 27-fold more sensitive to gemcitabine and 5-fold less sensitive to 5-FU than *Brca1*^+/+ ^MMECs. Interestingly, triple-negative breast cancers, which share a similar molecular and histopathological profile with *BRCA1*-mutated breast cancers, have also been found to be sensitive to gemcitabine [49,53]. Consistent with the mechanism of gemcitabine being independent of DNA repair, the increase in sensitivity to gemcitabine due to the loss of *Brca1 *expression was not a result of defective NER (Figure 4d) or double-strand break repair (Figure 4e). However, gemcitabine has been shown to induce H2AX phosphorylation and Rad51 nuclear foci formation, i.e. markers of DNA double strand breaks, at stalled replication forks in triple-negative breast cancer cells [49,54]. Therefore, it is possible that our experimental conditions for gemcitabine treatment did not produce significant double strand breaks. Both gemcitabine and 5-FU function as nucleoside analogs that inhibit DNA replication. However, gemcitabine also inhibits ribonucleotide reductase. This additional action of gemcitabine is likely to be responsible for the drastically different effects between the two drugs. For example, ribonucleotide reductase plays a role in maintaining the supply of dNTPS at sites of DNA damage to allow for efficient repair [55,56]. Gemcitabine-mediated inhibition of ribonucleotide reductase may preclude mechanisms of repair (other than NER or double-strand DNA break repair) from compensating in the absence of BRCA1-mediated DNA repair. Alternatively, functions other than DNA repair may determine gemcitabine sensitivity or 5-FU resistance in *Brca1*-deficient cells. These functions may be attributed to the RING-finger domain at the N-terminus of *BRCA1 *that functions in transcription regulation and/or the BRCT domain at the C-terminus of *BRCA1 *that functions in various processes through protein-protein interactions [43,44].

Loss of *Brca1 *does not affect the sensitivity to taxanes. Current reports on the involvement of BRCA1 in determining sensitivity to this class of drug are conflicting [41,57,58], and these data may be dependent on cell type or differences among model systems. Interestingly, a similar study using transformed *Brca1*^+/+ ^and *Brca1*^-/- ^mouse ovarian epithelial cell lines also showed no difference in sensitivity to paclitaxel [59]. Regardless, further studies are warranted to determine the exact role that BRCA1 and related proteins play in paclitaxel sensitivity (or resistance).

Cisplatin and gemcitabine exert a drastic synergistic effect on cellular sensitivity in the absence of BRCA1. We found that both *Brca1*^+/+ ^and *Brca1*^-/- ^MMECs showed synergy between the two drugs (Figure 3), and we and others have reported similar synergy in other cell types [49,53,60-63]. In fact, the cisplatin-gemcitabine combination is currently FDA-approved for use in the treatment of some non-small cell lung cancers. The relatively low concentrations that produced the individual drug sensitivities combined with the synergism in *Brca1*^-/- ^MMECs provides strong preclinical evidence for the cisplatin-gemcitabine combination in the treatment of *BRCA1*-associated breast cancers. While gemcitabine has not been shown to directly induce DNA damage (Figure 4e and [64]), nor has it shown dependency on DNA repair systems [65], it has been shown to inhibit repair of cisplatin-induced DNA damage [61,66,67], and this may contribute to the synergistic effect observed in *Brca1*-deficient cell lines. DNA repair is a mechanism of resistance to cisplatin [51]. Therefore, inhibition of repair, such as that due to gemcitabine, may produce sensitivity to cisplatin.

This study opens the door for the identification of other existing chemotherapeutic agents that may also be selectively sensitive to BRCA1-deficient cells. Other drugs may potentially be identified in a larger screen such as that described for BRCA2-deficient cells [68].

Due to the early success of PARP inhibitors, such as olaparib, in BRCA1-deficient tumors [10], it is possible that combination regimens including cisplatin and/or gemcitabine may be even more effective. In fact, we have recently initiated a Phase II clinical trial for gemcitabine, carboplatin and PARP inhibitor iniparib (BSI-201) in the neoadjuvant treatment of BRCA1 or BRCA2 mutated and triple-negative breast cancer.

Clinical evidence is emerging that BRCA1- associated breast cancers are particularly sensitive to platinum agents. A recent study using registry data from Poland identified 102 women who carried a *BRCA1 *founder mutation and had undergone neoadjuvant chemotherapy for breast cancer [69]. Remarkably, 83% (10 of 12) of those treated with cisplatin achieved a pathological complete response, compared to more typical rates of 22% with doxorubicin, cyclophosphamide and 5-FU based regimens. A report from the Dana-Farber Cancer Institute documented a 22% pathological complete response with single-agent cisplatin in the neoadjuvant setting for 28 women with early stage triple-negative breast cancer; only 2 patients were known *BRCA1 *mutation carriers, though both achieved a complete response [70]. At this point, nothing is known regarding gemcitabine as a selective targeted agent in *BRCA1 *mutant breast cancers.

## Conclusions

Doxorubicin, 5-FU, paclitaxel, and docetaxel are all currently used in breast cancer therapy. On the other hand, cisplatin and gemcitabine are not included in first line regimens for breast cancer, yet we found that they show therapeutic effectiveness in *Brca1*-deficient MMECs. Taken together, our data suggest a novel targeted approach to treating *BRCA1*-mutated or other DNA repair-deficient breast cancers to include gemcitabine and cisplatin. Based upon these results, clinical trials have been initiated to examine the role of platinum drugs with gemcitabine in *BRCA1*- mutant and triple-negative breast cancers [71].

## List of Abbreviations

6-4PP: 6-4 photoproduct; BER: base-excision repair; CPD: cyclobutane pyrimidine dimer; dNTPs: deoxyribonucleotides; GGR: global genomic repair; HR: homologous recombination; MMECs: murine mammary epithelial cells; MTT: 3-(4,5-dimethylthiazol-2-yl)-2,5-diphenyltetrazolium bromide; NER: nucleotide-excision repair; RT-qPCR: real-time reverse-transcriptase polymerase chain reaction; TCR: transcription-coupled repair; UV: ultraviolet.

## Authors' contributions

EA constructed the manuscript, carried out the analysis and interpretation of data, and generated the DNA strand break data. VBS participated in the design of the study and acquired the chemosensitivity data. ARH conducted the NER experiments. PSL and LM analyzed damage-induced expression of GGR genes. JMF conceived and supervised the study. All authors have read and approved the final manuscript.
